# Changes in disease burden and global inequalities in bladder, kidney and prostate cancers from 1990 to 2019: a comparative analysis based on the global burden of disease study 2019

**DOI:** 10.1186/s12889-024-18353-9

**Published:** 2024-03-25

**Authors:** Qiao Huang, Jun Yang, Guo-Xiong Liu, Hao Zi, Shi-Di Tang, Hai-Chang Jia, Wei Li, Xiao-Feng Xu, Xian-Tao Zeng

**Affiliations:** 1 Center for Evidence-Based and Translational Medicine, Zhongnan Hospital of Wuhan University, Wuhan, China; 2 Department of Urology, The First People’s Hospital of Tianmen in Hubei Province, The Affiliated Hospital of Hubei University of Science and Technology, Tianmen, China; 3 Department of Urology, Xianyang Central Hospital, Xianyang, China; 4 Department of Urology, Zhongnan Hospital of Wuhan University, Wuhan, China; 5 Department of Oncology, The First People’s Hospital of Tianmen in Hubei Province, The Affiliated Hospital of Hubei University of Science and Technology, Tianmen, China

**Keywords:** Burden of disease, Bladder cancer, Kidney cancer, Prostate cancer, Health inequality

## Abstract

**Background:**

Bladder, kidney and prostate cancers make significant contributors to cancer burdens. Exploring their cross-country inequalities may inform equitable strategies to meet the 17 sustainable development goals before 2030.

**Methods:**

We analyzed age-standardized disability-adjusted life-years (ASDALY) rates for the three cancers based on Global Burden of Diseases Study 2019. We quantified the inequalities using slope index of inequality (SII, absolute measure) and concentration index (relative measure) associated with national sociodemographic index.

**Results:**

Varied ASDALY rates were observed in the three cancers across 204 regions. The SII decreased from 35.15 (95% confidence interval, CI: 29.34 to 39.17) in 1990 to 15.81 (95% CI: 7.99 to 21.79) in 2019 for bladder cancers, from 78.94 (95% CI: 75.97 to 81.31) in 1990 to 59.79 (95% CI: 55.32 to 63.83) in 2019 for kidney cancer, and from 192.27 (95% CI: 137.00 to 241.05) in 1990 to − 103.99 (95% CI: − 183.82 to 51.75) in 2019 for prostate cancer. Moreover, the concentration index changed from 12.44 (95% CI, 11.86 to 12.74) in 1990 to 15.72 (95% CI, 15.14 to 16.01) in 2019 for bladder cancer, from 33.88 (95% CI: 33.35 to 34.17) in 1990 to 31.13 (95% CI: 30.36 to 31.43) in 2019 for kidney cancer, and from 14.61 (95% CI: 13.89 to 14.84) in 1990 to 5.89 (95% CI: 5.16 to 6.26) in 2019 for prostate cancer. Notably, the males presented higher inequality than females in both bladder and kidney cancer from 1990 to 2019.

**Conclusions:**

Different patterns of inequality were observed in the three cancers, necessitating tailored national cancer control strategies to mitigate disparities. Priority interventions for bladder and kidney cancer should target higher socioeconomic regions, whereas interventions for prostate cancer should prioritize the lowest socioeconomic regions. Additionally, addressing higher inequality in males requires more intensive interventions among males from higher socioeconomic regions.

**Supplementary Information:**

The online version contains supplementary material available at 10.1186/s12889-024-18353-9.

## Introduction

Cancer is a significant public health issue worldwide [1]. Genitourinary cancer generally refers to cancers of the urinary system of men and women and reproductive organs in men [2]. Prostate, bladder and kidney cancers are the most common histological types of genitourinary cancers. In cancer statistics for 2020, prostate cancer stood as the second most prevalent cancer and the fifth leading cause of cancer-related mortality among men globally [1]. Meanwhile, bladder and kidney cancer rank as the 10th and 14th most common cancer worldwide [1, 3]. Both morbidity and mortality rates of the three cancers are predicted to increase in next decade [4]. The three cancers make significant contributions to the global cancer burden and deserve to be studied [4, 5]. Disability-adjusted life years (DALYs) is a crucial and comprehensive metric used to quantify the loss of years of healthy life due to illness, disability or early death. The DALYs for cancer come from years of life lost due to high premature mortality (YLLs) and years lived with disability (YLDs) among cancer survivors through diagnoses, side effects of treatment, and living with cancer as a chronic disease [6]. Prostate cancer leads to the highest disease burden globally with 8644.87 × 10^3^ DALYs (only male), followed by 4392.58 × 10^3^ DALYs for bladder cancer (both male and female) and 4052.82 × 10^3^ DALYs for kidney cancer (both male and female) [5]. Reducing DALYs due to the three cancers requires public health professionals prioritizing interventions and allocating resources effectively.

The total DALYs caused by cancers varied by countries. In 2013, the DALYs caused by cancer in China were 10 times higher than the DALYs caused by cancer in Japan [7]. More developed countries had a higher proportion of DALYs due to YLDs compared to less developed countries where premature mortality (YLLs) dominated [6]. Premature deaths from the top five cancers were significantly higher in countries with a higher human development index (HDI) [8]. The uneven distribution of health loss from cancers across populations is the inequality of disease burden [9]. Ignoring inequality means health policy and resources, funding and interventions are not preferentially targeted to populations that need them most. High disease burden will persist in disadvantaged regions and demographic groups, which further widen the health and economic gap and exacerbate global instability. Globally, countries with higher socioeconomic development had greater disease burden and higher mortality rates but also showed greater declines over time [5]. These uneven patterns highlight a need to comprehensively assess cross-country inequality in the global genitourinary cancer burden. Previous studies have assessed the inequality burden of communicable and non-communicable diseases [10–12]. However, few studies have used using scientific measures to quantitatively and comprehensively assessed cross-country inequalities in prostate, bladder and kidney cancers. Based on the high and increasing DALYs of the three cancers, it is of great significance to assess their inequality geographically and track the trajectory of inequality over time for future public health interventions.

The Age-standardized DALY (ASDALY) rate allows comparisons across different regions by accounting for variations in population and age distributions. In this study, we extracted ASDALY rates by cancer type (prostate, bladder and kidney) and sex (male, female and both) across 204 regions from the Global Burden of Diseases, Injuries, and Risk Factors Study (GBD) 2019 [13]. Absolute inequality measures the absolute difference in health outcomes between the most advantaged and most disadvantaged groups. Relative inequality can quantify the extent to which health outcomes are concentrated across groups with different development levels [14]. In our study, we utilized the slope index of inequality (SII) to quantify absolute inequality and the Concentration Index to quantify relative inequality, both of which are recommended by the World Health Organization (WHO) [15]. These indices were applied to assess the global-scale inequalities in prostate, bladder, and kidney cancers from 1990 to 2019 across 204 regions with different levels of socioeconomic development. Additionally, considering the multifaceted biological, social, cultural, and economic differences between males and females, we further conducted separate assessment of global inequality using male and female data. By understanding the global-scale inequalities and comparing the inequalities across different cancer types and by sex, our research aims to provide evidences for identifying vulnerable and disadvantaged groups that experience a disproportionate burden of disease. It will inform cross-national learning and establish priorities for interventions and policies, finally help realize the 10th goal (reduce inequality) of the 17 sustainable development goals (SDGs) by 2030.

## Methods

### Data source

The GBD study is a comprehensive and systematic database that provides a valuable overview of disease burden on a global scale. It can serve as a valuable resource for policymakers, researchers, and the general public to understand health challenges and track progress over time. The database quantifies the comparative magnitude of health loss for hundreds of diseases, injuries, and risk factors across approximately 204 countries and territories from 1990 and 2019. The latest GBD study 2019 was released in 2020, in which a Bayesian meta-regression based DisMod-MR 2.1 was used to pool different data sources [16]. DALYs were computed by combining YLLs and YLDs. YLDs were calculated by multiplying the prevalence of specific health conditions with disability weights. YLLs were determined by multiplying cause-specific mortality rates with the remaining years expected at the time of death, based on a reference life expectancy. Based on the GBD study’s world population age standard, the ASDALY per 100,000 individuals was estimated facilitating comparisons. The 95% uncertainty intervals (UIs) for these estimates were derived from the 25th and 975th percentiles of 1000 draws from the posterior distribution.

In this study, we used the online Global Health Data Exchange query tool to extract the ASDALY rate and its 95% UI by nations, cancers (prostate, bladder, kidney cancer), sex (male and female) and age group (age-standardized rate). Additionally, we extracted national population figures and the sociodemographic index (SDI) from the GBD study 2019 database. The SDI quantifies a region or country’s socio-demographic development level. Summary and public information were used and analyzed in this study, so no ethical approval was required.

### Statistical analysis

To assess the distribution of health outcomes across different socioeconomic groups, two measures of SDI-related inequalities were used: the SII and the concentration index. The SII serves as an absolute measure, representing the differences in average health outcomes between the top and bottom socioeconomic groups [17]. To calculate the SII, the 204 nations and territories were firstly ranked by SDI, and a relative rank was defined as the midpoint of the cumulative distribution of the total population, ranging from 0 (the bottom group) to 1 (the top group). A weighted least squared regression was performed by regressing ASDALY rate of 204 countries and territories on their relative ranks using the population size as the weight. A square root transformation was applied to the regression to account for potential heteroscedasticity, giving a hypothetic formula $\left(\textrm{ASDALY}\ \textrm{rate}\right)\times \sqrt{S}\sim {\beta}_0\times \sqrt{S}+{\beta}_1\times \textrm{R}\times \sqrt{S}$, where S is the proportion of population size in the total population and R is the relative rank. The *β*_1_ was estimated as the SII, its point estimate and 95% confidence interval (CI) have been reported [9].

The Concentration Index serves as a relative measure for inequality, with higher values indicating greater inequality [18]. A concentration curve was created by plotting the cumulative distribution of ASDALY rate (Y axis) against the cumulative distribution of the population, ranked based on the SDI level (X axis). The 45-degree line, also known as the line of equality, represents perfect equality where cancer burden is distributed equally across different regions regardless of their position on the SDI levels. Meanwhile, a concentration curve below the line of equality indicates that the cancer burden is more concentrated among regions with higher SDI. The area between the concentration curve and the line of equality was trapezoidally integrated, and the Concentration Index was equal to twice the area. Corresponding 95% CI was estimated using a bias-corrected bootstrap method with 2000 replicates.

In this study, we have firstly provided the national description and visualization of ASDALY rates for prostate, bladder, kidney cancers in 1990 and 2019, along with their changes. Another plot was then generated to illustrate the distributions of ASDALY rates for the three cancers in 2019, ranging from regions with the highest SDI to those with the lowest SDI. Secondly, we calculated both the SII and Concentration Index for the three cancers. A statistically significant inequality measure is indicated by its 95% confidence intervals that do not include zero. Lastly, to compare the inequality of cancer burden between males and females, we conducted a separate assessment of inequality by sex (female and male data). When comparing two inequality measures, a statistically significant difference is indicated when the 95% CIs of the two measures do not overlap [19]. All statistical analyses and data visualization were performed using the R program (version 4.0.3, R core team, Vienna, Austria) with PHEindicatormethods (version 2.0.1), pracma (version 2.4.2), boot (version 1.3–28.1), ggplot2 (version 3.4.3), cowplot (version 1.1.1) and circlize (Version 0.4.15) packages.

## Results

### Bladder cancer

The ASDALY rate of bladder cancer demonstrated significant variation across the 204 countries and territories in both 1990 and 2019 see Supplementary Fig. 1. In 1990, Lebanon had the highest ASDALY rate at 225.45 (95% UI, 184.83, 274.45), and El Salvador had the lowest at 18.94 (95% UI, 17.47, 20.49). Similarly, in 2019, Egypt reported the highest ASDALY rate at 201.75 (95% UI, 132.14, 294.39), while Albania reported the lowest at 20.70 (95% UI, 15.62, 27.07). Cabo Verde experienced the most significant increase in ASDALY rate, with a rise of 42.55, whereas Bahrain exhibited the highest decrease, with a decline of 62.78.

**Fig. 1:**
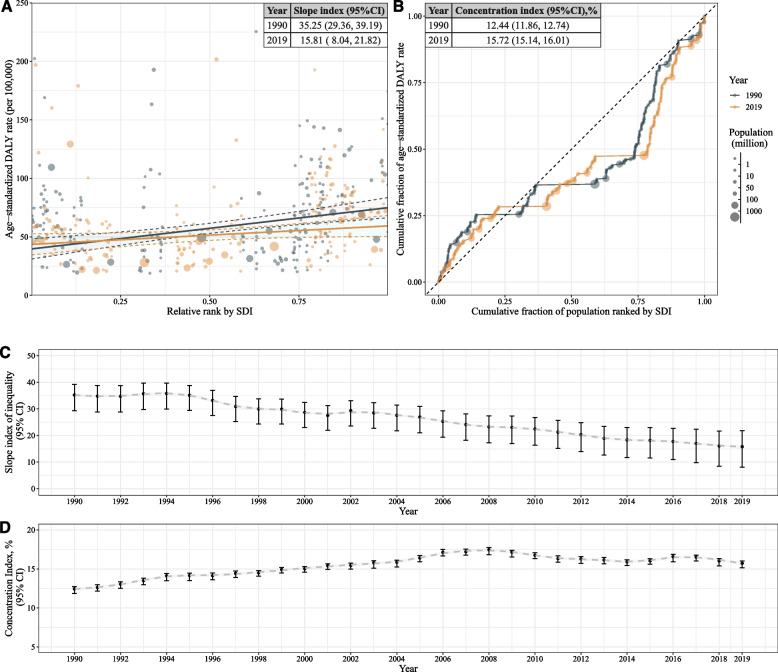
Inequality of bladder cancer burden from 1990 to 2019. **A** Scatter plot of age standardized DALYs rates and Slope index of inequality in 1990 and 2019; (**B**) Lorenz curve and Concentration index in 1990 and 2019; (**C**) Change of slope index of inequality from 1990 to 2019; (**D**) Change of concentration index from 1990 to 2019

The assessment of inequalities in the burden of bladder cancer (Fig. 1) revealed notable absolute and relative SDI-related inequalities in all populations. Countries with a higher SDI displayed a considerably greater burden. We observed a continuous decrease in SII from 35.15 (95% CI: 29.34 to 39.17) in 1990 to 15.81 (95% CI: 7.99 to 21.79) in 2019 (see Fig. 1 A and C). Supplementary Fig. 2 (A) and Fig. 3 (A) showed the difference in ASDALY rates between males and females was smaller in lower socioeconomic regions compared to higher socioeconomic regions. Supplementary Fig. 2 (A and C) showed a higher SII in male group, where it fell from 5.22(95% CI: 84.67, 101.73) in 1990 to 48.01 (95% CI:32.46, 59.04) in 2019. However, in female group, the SII was 3.24 (95% CI: − 1.98, 6.21) in 1990, which suggested no absolute inequality. It further shifted to − 5.57 (95% CI: − 11.31, − 2.21) in 2019 (Supplementary Fig. 3 A and C). An upward trend in the concentration index was noted, with values of 12.44 (95% CI, 11.86 to 12.74) in 1990 and 15.72 (95% CI, 15.14 to 16.01) in 2019 (Fig. 1B and D). The relative inequality was mainly attributable to the male group which remained stable from 1990 to 2019 (Supplementary Fig. 2 B and D). For the female group, the concentration index was low but showed an upward trend, increasing from 1 1.76 (95%CI: 1.13, 2.02) in 1990 to 5.72 (95%CI:5.07, 6.01) in 2019 (Supplementary Fig. 3 B and D).

### Kidney cancer

Significant variations in the ASDALY rate related to kidney cancer were observed across 204 countries and territories in both 1990 and 2019, see Supplementary Fig. 4. During 1990, Uruguay reported the highest ASDALY rate at 173.07 (95% UI: 164.18, 182.47), while Kenya had the lowest at 10.50 (95% UI: 6.56, 13.15). Similarly, in 2019, Uruguay maintained its position with the highest ASDALY rate at 166.63 (95% UI: 149.92, 184.54), with Papua New Guinea having the lowest at 14.81 (95% UI: 10.48, 21.64). Belarus experienced a substantial increase in ASDALY rate, rising by 82.44, while Saint Kitts and Nevis exhibited the biggest decrease, declining by 61.71.

**Fig. 2:**
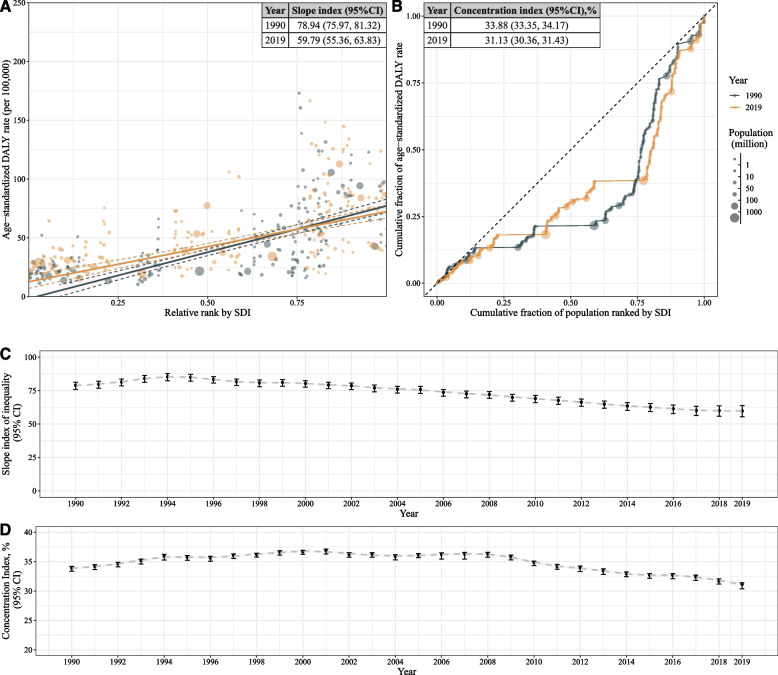
Inequality of kidney cancer burden from 1990 to 2019. **A** Scatter plot of age standardized DALYs rates and Slope index of inequality in 1990 and 2019; (**B**) Lorenz curve and Concentration index in 1990 and 2019; (**C**) Change of slope index of inequality from 1990 to 2019; (**D**) Change of concentration index from 1990 to 2019

A high and significant inequality was observed in the burden of kidney cancer across countries (Fig. 2). The SII exhibited a slightly decreasing trend from 1990 to 1994, followed by a continuous decline until 2019, with an SII of 78.94 (95% CI: 75.97 to 81.31) in 1990 and 59.79 (95% CI: 55.32 to 63.83) in 2019 (Fig. 1 A and C). Similar to bladder cancer, see Supplementary Fig. 5 (A) and Fig. 6 (A), ASDALY rate differences between males and females were smaller in lower socioeconomic regions compared to higher ones. The absolute inequality was mainly attributable to the male group. In male group, the SII decreased from 112.49 (107.17,116.36) in 1990 to 92.72 (84.74,99.88) in 2019 (Supplementary Fig. 5 A and C). Meanwhile, in the female group, the SII decreased from 53.35 (51.30,55.03) in 1990 to 31.24 (95% CI: 27.88,34.08) in 2019 (Supplementary Fig. 6 A and C). The Concentration Index exhibited a slightly increasing trend from 1990 to 1994, it then remained stable from 1994 to 2008 and began to decline after 2008. The concentration index was 33.88 (95% CI: 33.35 to 34.17) in 1990 and 31.13 (95% CI: 30.36 to 31.43) in 2019 (Fig. 2 B and D). Males and Females shared similar relative inequality and showed decreasing trend. For males, the concentration index was 36.03 in 1990 and 33.33 in 2019 (Supplementary Fig. 5 B and D). For females, the concentration index was 32.27 in 1990 and 26.72 in 2019 (Supplementary Fig. 6 B and D).

### Prostate cancer

High level and remarkable variation in the ASDALY rate related to prostate cancer were observed across the 204 countries and territories in both 1990 and 2019, seen in Supplementary Fig. 7. In 1990, Dominica reported the highest ASDALY rate at 1591.53 (95% UI: 1358.78, 2061.22), while Egypt reported the lowest at 105.13 (95% UI: 89.53, 135.24). In 2019, Dominica maintained its position with the highest ASDALY rate of 1923.95 (95% UI: 1428.24, 2394.31), while Bangladesh documented the lowest ASDALY rate at 118.34 (95% UI: 68.94, 181.71). Moreover, we observed great change in ASDALY rate from 1990 to 2019. The largest increase was documented in Cabo Verde at 758.96, while the Switzerland reported the highest decrease at 267.82.

**Fig. 3:**
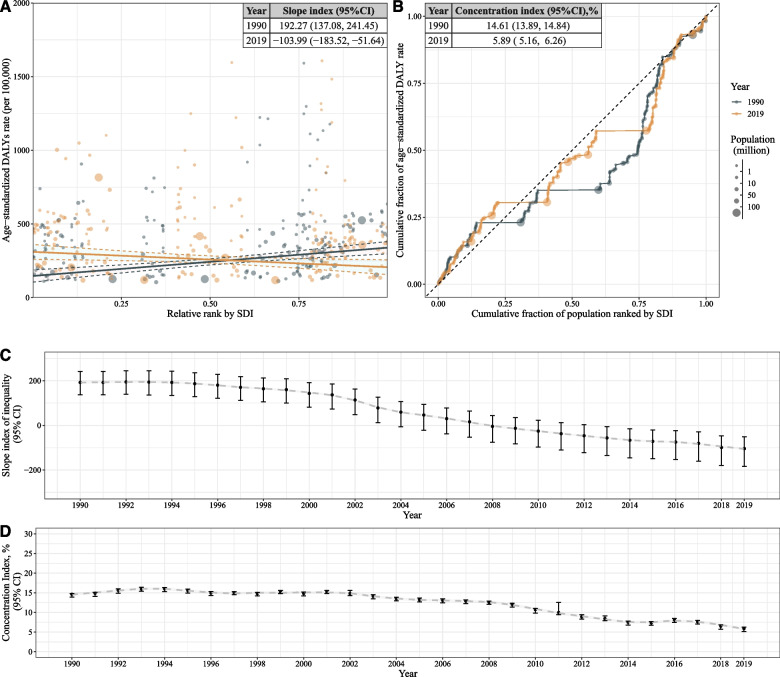
Inequality of prostate cancer burden from 1990 to 2019. **A** Scatter plot of age standardized DALYs rates and Slope index of inequality in 1990 and 2019; (**B**) Lorenz curve and Concentration index in 1990 and 2019; (**C**) Change of slope index of inequality from 1990 to 2019; (**D**) Change of concentration index from 1990 to 2019

Despite the high ASDALY rate of prostate cancer, a significant reduction in inequality was observed in the global burden of prostate cancer (Fig. 3). We observed a continuous decrease in SII from 192.27 (95% CI: 137.00 to 241.05) in 1990 to − 103.99 (95% CI: − 183.82 to 51.75) in 2019 (Fig. 3A and C). Meanwhile, the concentration index also decreased, from 14.61 (95% CI: 13.89 to 14.84) in 1990 to 5.89 (95% CI: 5.16 to 6.26) in 2019 (Fig. 3B and D).

## Discussion

In this study, we utilized the worldwide GBD study 2019 data to assess cross-country inequality for the bladder, kidney and prostate cancers from 1990 to 2019. Absolute inequalities were decreasing in all three cancers from 1990 to 2019, in which the SII of prostate cancer changed from positive to negative. In 2019, the positive SIIs were documented for bladder and kidney cancer, which suggested regions with the highest socioeconomic level continued to bear higher cancer burdens compared to regions with the lowest socioeconomic level. Conversely, the negative SII in prostate cancer suggested higher burdens in regions with the lowest socioeconomic levels. The greatest relative inequality was noted in kidney cancer from 1990 to 2019, followed by bladder cancer and prostate cancer. Heterogeneity in change of relative inequality from 1990 to 2019 was observed. Both prostate and kidney cancers exhibited decreased relative inequality, while bladder cancer showed a slight increase. Moreover, there was a notable disparity in inequality between male and female groups in bladder and kidney cancers, with the male group exhibiting higher inequality than females. Tailoring interventions based on the observed disparities can contribute to reducing the burden of cancer and promoting more equitable outcomes of healthcare.

The three genitourinary cancers reported positive SII and Concentration Index in about 20 years. It indicated that the disease burdens are more prevalent and concentrated among the countries with higher SDI. A study on GBD study database reported that countries with higher SDI quintile gave higher incident cases and higher age-standardized incidence rate in kidney, bladder, and prostate cancers [5]. Countries with higher SDI have better healthcare and education, which results in aging populations in these countries [20]. Generally, cancer incidence tends to increase with age. Urbanization and industrialization in high SDI countries can lead to unhealthy lifestyle changes, such as unhealthy diets and reduced physical activity [21]. Meanwhile, the risk of exposure to environmental pollutants or specific carcinogens increased significantly. For example, a higher prevalence of tobacco use was observed in high-SDI countries [22]. Arsenic poisoning occurs most often in areas having major industrialization and a systematic review has showed that exposure to arsenic in drinking water increases the risk of kidney cancer [23]. These factors can lead to positive SII and concentration index among the three genitourinary cancers. In recent years, global public health interventions have been conducted to improve healthcare access and quality, especially in lower socioeconomic groups [24]. From 1990 to 2019, both the SII and concentration index for the three cancers decreased, except for the concentration index of bladder cancer, which showed a slight increase. Although the difference in bladder cancer burden between the highest and the lowest socioeconomic groups has narrowed, the cancer burden has become more concentrated among the higher socioeconomic groups. This may be attributed to the increased prevalence of bladder cancer-specific risk factors in higher socioeconomic groups, such as occupational exposures to aromatic amines and polycyclic aromatic hydrocarbons [25]. Further research is required to explore the underlying mechanisms for bladder cancer, which will guide effective and equal public health and prevention strategies. Our study found that kidney cancer had the highest inequality among the three cancers since 2008 with multiple factors potentially contributing to this. Advancements in diagnostic techniques and imaging technologies, including prostate-specific antigen (PSA) based screening and cystoscopy have improved early detection rates for prostate and bladder cancers [26]. However, these advancements might not be equally accessible or widely implemented for kidney cancer. Meanwhile, early symptoms of kidney cancer are often insidious and may not have obvious symptoms, making early diagnosis relatively difficult [27]. Moreover, some populations or socio-economic groups may have a higher prevalence of exposure to specific risk factors, such as obesity and hypertension, which are more common in high SDI countries [28]. These factors might lead to disparities in detecting and treating the disease at an early stage. Nevertheless, further research and analysis would be necessary to understand the specific drivers of this disparity. Public health efforts should focus on improving early detection and treatment of kidney cancer.

A negative SII was observed in prostate cancer since 2008. In low SDI countries, access to high-quality healthcare services may be limited, including PSA screening, early detection and advanced treatments. Prostate cancer would therefore have gone undiagnosed or have been diagnosed at later stages, leading to aggressive prostate cancer that was harder to treat resulting in a higher mortality rate [29]. Consequently, the disease burden of prostate cancer is disproportionately concentrated in low SDI countries, despite its relatively low incidence in those regions. By tailoring interventions to different countries, policymakers will effectively reduce the disparities in disease burden between low and high SDI countries.

Burdens of bladder and kidney cancers were more unequally distributed among males than females globally. Overall, regions with higher SDI and male population exhibited higher incidence, mortality, and DALYs for bladder and kidney cancers [5]. Our findings indicated that the difference in ASDALY rates between males and females primarily originates from higher socioeconomic regions. In more highly development regions, males may be more prone to engaging in risky behaviors, such as smoking and heavy alcohol consumption, which are known risk factors of bladder and kidney cancers [30, 31]. High levels of development are associated with high industrial development. Industries with higher male participation may involve greater exposure to occupational hazards linked to bladder and kidney cancer. Areas with more industrial activity reported higher rates of bladder and kidney cancer [32]. While both males and females in higher socioeconomic regions have better access to healthcare, there may still be gender disparities in the utilization of these services. Men might be less likely to seek preventive care or early detection, leading to a higher burden of disease after diagnosis [33]. Meanwhile, previous studies showed that males might experience poorer survival outcomes in bladder and kidney cancers than females [34, 35]. Therefore, gender-specific preventive and therapeutic approaches are essential to optimize gender health equality for all.

The projected rise in the three cancers over the coming decade highlights a concerning trend in future health burdens [4]. Diverse model of inequalities found in three cancers holds immense significance for future tailored public health strategies. Vital strategies include promoting preventive measures, advocating regular screenings, raising awareness about symptoms, and enhancing access to diagnostic and treatment resources [36]. Based on the distribution of ASDALY rates and corresponding SDI (Supplementary Fig. 8), regions with lower cancer burden could offer insights into effective interventions for regions with higher burdens, as seen in examples like Albania compared to Egypt in bladder cancer, Papua New Guinea compared to Uruguay in kidney cancer, and Bangladesh compared to Dominica in prostate cancer. Fostering partnerships, international organizations, and non-governmental entities is crucial for enabling the exchange of knowledge, building capacities, and effectively implementing best practices. Ultimately, these collective endeavors will significantly contribute to advancing global health equity for genitourinary cancers.

This study has systematically assessed the inequality and its trend in three genitourinary cancers. The noted disproportionate burden carried substantial implications for public health. Nonetheless, there were several limitations in this study. Firstly, complex statistical models were used to model data and estimate metrics in GBD study, underdeveloped countries may underestimate figures due to missed diagnosis and low registration, etc. The accuracy of our analysis depended on accuracy of available data reported in GBD study database. Secondly, even though trend of inequality from 1990 and 2019 was assessed, the GBD study 2021 will be released in the future and this can provide a more accurate estimation. Meanwhile, the COVID-19 epidemic since 2020 has had a great effect on both economic systems and medical development, its impact on inequality of disease burden can be further assessed. Lastly, recent advances in screening, diagnosis and treatment will impact the results, which might limit the generalization of our findings.

## Conclusion

Our study has revealed a complex pattern of inequalities from 1990 to 2019 in the burden of bladder, kidney, and prostate cancers across different socioeconomic levels. It can inform evidence-based priorities for customized interventions and policies to realize the 10th goal (reduce inequality) by 2030. From 2019, regions with higher sociodemographic status should focus on reducing bladder and kidney cancer-specific risk factors. Conversely, for prostate cancer, regions with the lowest sociodemographic status should prioritize the provision of high-quality healthcare services to ensure early detection and timely treatments. Higher inequality in males was observed for bladder and kidney cancer, more intensive interventions should be focused on males from higher socioeconomic regions, such as improving awareness reducing risk behaviors and increasing protective measures during industrial activity. International cooperation is vital to promote equitable healthcare outcomes and reshape the landscape of global health.

### Supplementary Information

**Supplementary Material 1.**

## Data Availability

Data are available in a public, open access repository with free access (https://vizhub.healthdata.org/gbd-results/). The R code for statistical analysis of this study are available upon request from the first author and corresponding author.
