# Supplementary material for: Changes in disease burden and global inequalities in bladder, kidney and prostate cancers from 1990 to 2019: a comparative analysis based on the global burden of disease study 2019
Source: BMC Public Health. 2024 Mar 25;24:891. doi: 10.1186/s12889-024-18353-9 (PMC10962085; doi:10.1186/s12889-024-18353-9)
Supplement: Supplementary file 1 — Supplementary Material 1. [file 12889_2024_18353_MOESM1_ESM.pdf]

Legends of supplementary figures

(1) Supplementary figure 1 Age standardized DALYs rates of bladder cancer in 1990, 2019 and change from 1990 to 2019. (A) Change in age standardized DALYs rates from 1990 to 2019; (B) Age standardized DALYs rates in 2019; (C) Age standardized DALYs rates in 1990;

(2) Supplementary figure 2 Inequality of bladder cancer burden in males from 1990 to 2019. (A) Scatter plot of age standardized DALYs rates and Slope index of inequality in 1990 and 2019; (B)Lorenz curve and Concentration index in 1990 and 2019; (C) Change of slope index of inequality from 1990 to 2019; (D) Change of concentration index from 1990 to 2019;

(3) Supplementary figure 3 Inequality of bladder cancer burden in females from 1990 to 2019. (A) Scatter plot of age standardized DALYs rates and Slope index of inequality in 1990 and 2019; (B)Lorenz curve and Concentration index in 1990 and 2019; (C) Change of slope index of inequality from 1990 to 2019; (D) Change of concentration index from 1990 to 2019;

(4) Supplementary figure 4 Age standardized DALYs rates of kidney cancer in 1990, 2019 and change from 1990 to 2019. (A) Change in age standardized DALYs rates from 1990 to 2019; (B) Age standardized DALYs rates in 2019; (C) Age standardized DALYs rates in 1990;

(5) Supplementary figure 5 Inequality of kidney cancer burden in males from 1990 to 2019. (A) Scatter plot of age standardized DALYs rates and Slope index of inequality in 1990 and 2019; (B)Lorenz curve and Concentration index in 1990 and 2019; (C) Change of slope index of inequality from 1990 to 2019; (D) Change of concentration index from 1990 to 2019;

(6) Supplementary figure 6 Inequality of kidney cancer burden in females from 1990 to 2019. (A) Scatter plot of age standardized DALYs rates and Slope index of inequality in 1990 and 2019;

(B)Lorenz curve and Concentration index in 1990 and 2019; (C) Change of slope index of inequality from 1990 to 2019; (D) Change of concentration index from 1990 to 2019;

(7) Supplementary figure 7 Age standardized DALYs rates of prostate cancer in 1990, 2019 and change from 1990 to 2019. (A) Change in age standardized DALYs rates from 1990 to 2019; (B) Age standardized DALYs rates in 2019; (C) Age standardized DALYs rates in 1990;

(8) Supplementary figure 8 Age standardized DALYs rates of bladder, kidney and prostate cancer in 2019 along with sociodemographic index. (A) Sociodemographic index; (B) Age-standardized DALY rate of bladder cancer; (C) Age-standardized DALY rate of kidney cancer; (D)Age-standardized DALY rate of prostate cancer;

Supplementary figure 1 Age standardized DALYs rates of bladder cancer in 1990, 2019 and change from 1990 to 2019. (A) Change in age standardized DALYs rates from 1990 to 2019; (B) Age standardized DALYs rates in 2019; (C) Age standardized DALYs rates in 1990;

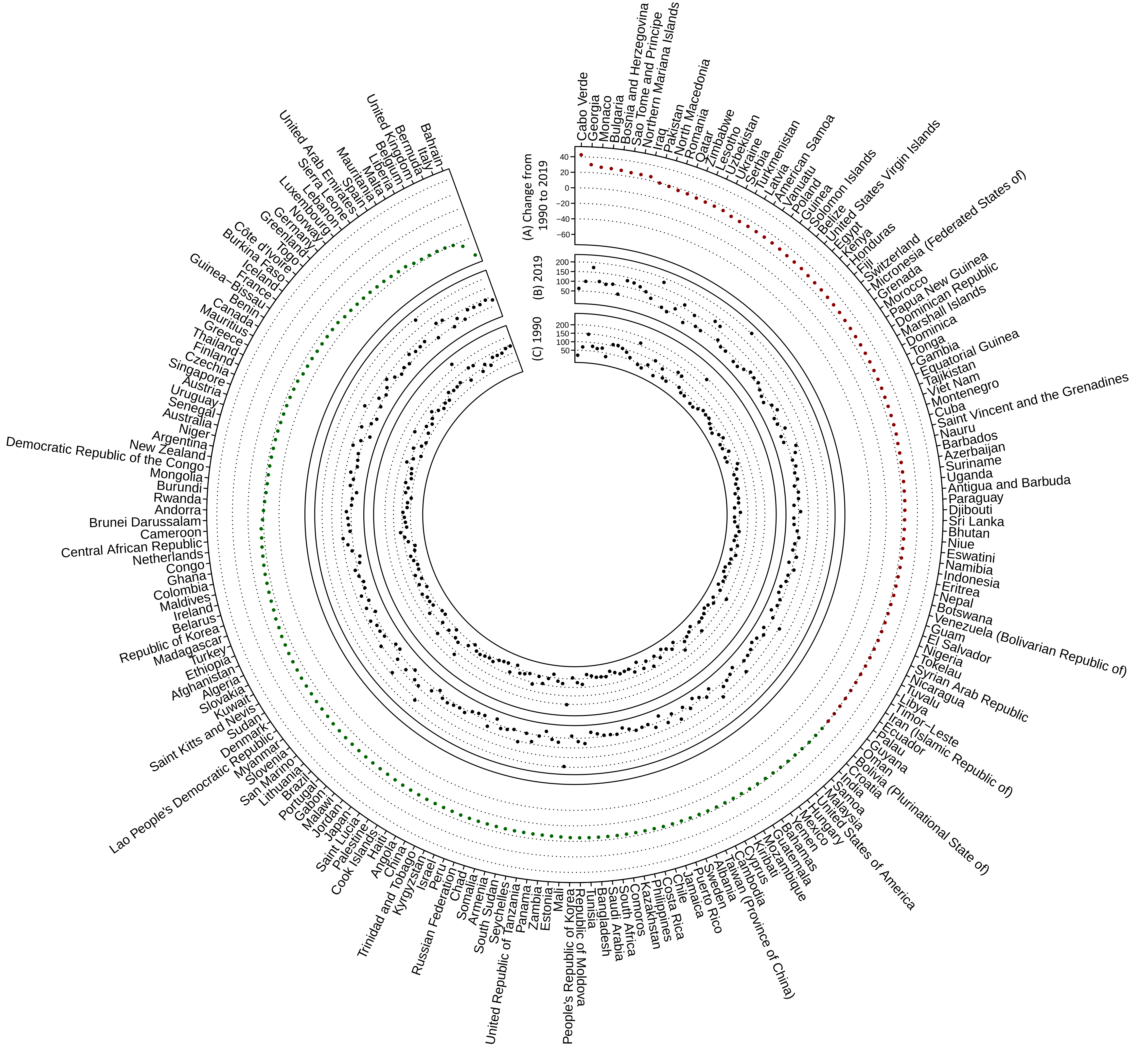
(A) Change from 1990 to 2019
40
20
0
-20
-40
-60
(B) 2019
200
150
100
50
(C) 1990
200
150
100
50
Cabo Verde
Georgia
Monaco
Bulgaria
Bosnia and Herzegovina
Sao Tome and Principe
Northern Mariana Islands
Iraq
Pakistan
North Macedonia
Romania
Qatar
Zimbabwe
Lesotho
Uzbekistan
Ukraine
Serbia
Turkmenistan
Latvia
American Samoa
Vanuatu
Poland
Guinea
Solomon Islands
Belize
United States Virgin Islands
Egypt
Kenya
Honduras
Fiji
Switzerland
Micronesia (Federated States of)
Grenada
Morocco
Papua New Guinea
Dominican Republic
Marshall Islands
Dominica
Tonga
Gambia
Equatorial Guinea
Tajikistan
Viet Nam
Montenegro
Cuba
Saint Vincent and the Grenadines
Nauru
Barbados
Azerbaijan
Suriname
Uganda
Antigua and Barbuda
Paraguay
Djibouti
Sri Lanka
Bhutan
Niue
Eswatini
Namibia
Indonesia
Eritrea
Nepal
Botswana
Venezuela (Bolivarian Republic of)
Guam
El Salvador
Nigeria
Tokelau
Syrian Arab Republic
Nicaragua
Tuvalu
Libya
Timor-Leste
Iran (Islamic Republic of)
Ecuador
Palau
Guyana
Oman
Bolivia (Plurinational State of)
Croatia
India
Samoa
Malaysia
United States of America
Hungary
Mexico
Yemen
Bahamas
Guatemala
Mozambique
Kiribati
Cyprus
Cambodia
Taiwan (Province of China)
Albania
Sweden
Puerto Rico
Jamaica
Chile
Costa Rica
Philippines
Kazakhstan
Comoros
South Africa
Saudi Arabia
Bangladesh
Tunisia
Republic of Moldova
People's Republic of Korea
Mali
Estonia
Zambia
Panama
United Republic of Tanzania
Seychelles
South Sudan
Armenia
Somalia
Chad
Russian Federation
Peru
Israel
Kyrgyzstan
Trinidad and Tobago
China
Angola
Haiti
Cook Islands
Palestine
Saint Lucia
Japan
Jordan
Malawi
Gabon
Portugal
Brazil
Lithuania
San Marino
Slovenia
Myanmar
Lao People's Democratic Republic
Denmark
Sudan
Saint Kitts and Nevis
Kuwait
Slovakia
Algeria
Afghanistan
Ethiopia
Turkey
Madagascar
Republic of Korea
Belarus
Ireland
Maldives
Colombia
Ghana
Congo
Netherlands
Central African Republic
Cameroon
Brunei Darussalam
Andorra
Rwanda
Burundi
Mongolia
Democratic Republic of the Congo
New Zealand
Argentina
Niger
Australia
Senegal
Uruguay
Austria
Singapore
Czechia
Finland
Thailand
Greece
Mauritius
Canada
Benin
Guinea-Bissau
France
Iceland
Burkina Faso
Côte d'Ivoire
Togo
Greenland
Germany
Norway
Luxembourg
Lebanon
Sierra Leone
United Arab Emirates
Spain
Mauritania
Malta
Liberia
Belgium
United Kingdom
Bermuda
Italy
Bahrain

Supplementary figure 2 Inequality of bladder cancer burden in males from 1990 to 2019. (A) Scatter plot of age standardized DALYs rates and Slope index of inequality in 1990 and 2019; (B)Lorenz curve and Concentration index in 1990 and 2019; (C) Change of slope index of inequality from 1990 to 2019; (D) Change of concentration index from 1990 to 2019;

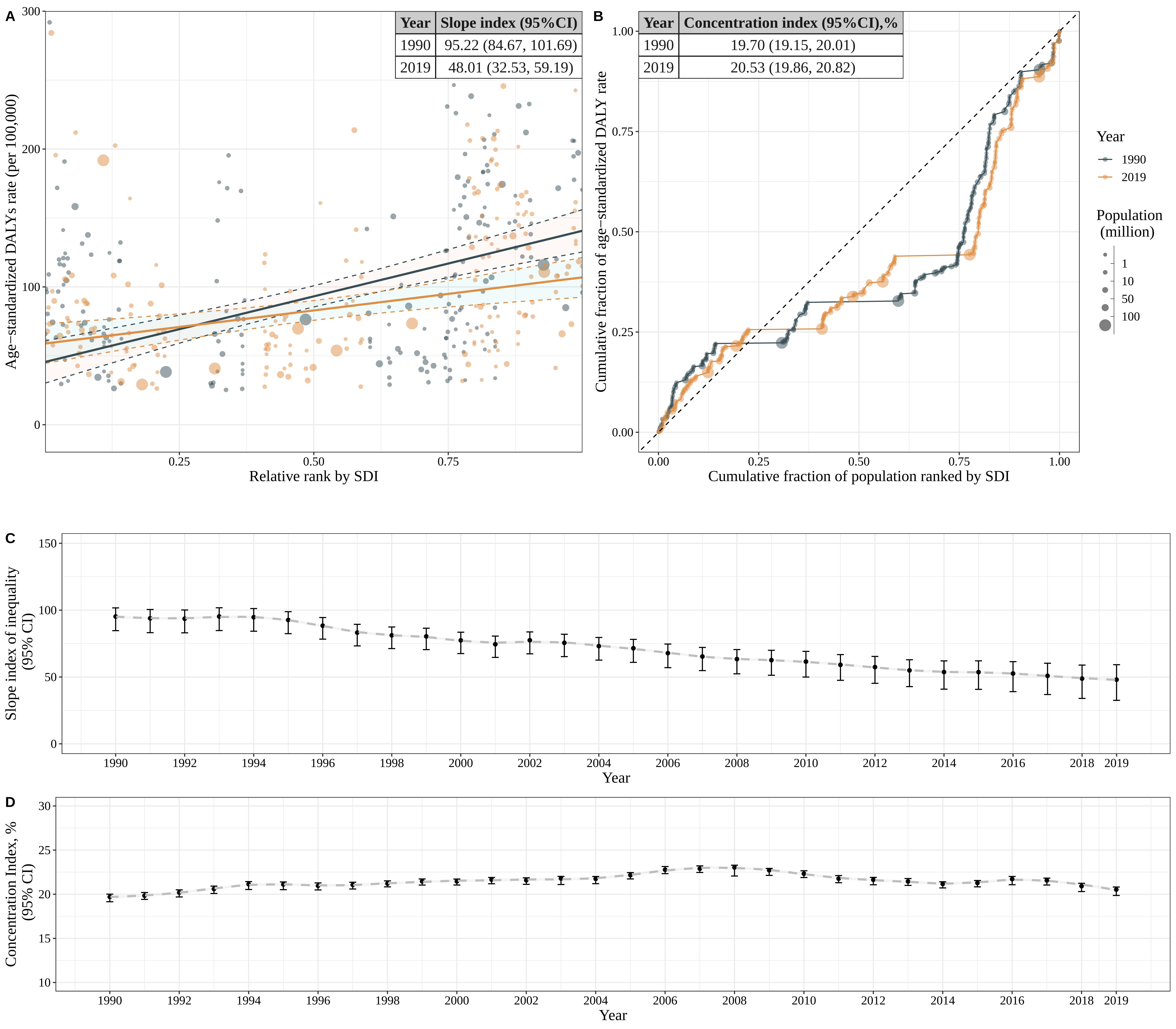
A
Year | Slope index (95%CI)
1990 | 95.22 (84.67, 101.69)
2019 | 48.01 (32.53, 59.19)
Age−standardized DALYs rate (per 100,000)
Relative rank by SDI
B
Year | Concentration index (95%CI),%
1990 | 19.70 (19.15, 20.01)
2019 | 20.53 (19.86, 20.82)
Cumulative fraction of age−standardized DALY rate
Cumulative fraction of population ranked by SDI
Year
1990
2019
Population (million)
1
10
50
100
C
Slope index of inequality (95% CI)
Year
D
Concentration Index, % (95% CI)
Year

Supplementary figure 3 Inequality of bladder cancer burden in females from 1990 to 2019. (A) Scatter plot of age standardized DALYs rates and Slope index of inequality in 1990 and 2019; (B)Lorenz curve and Concentration index in 1990 and 2019; (C) Change of slope index of inequality from 1990 to 2019; (D) Change of concentration index from 1990 to 2019;

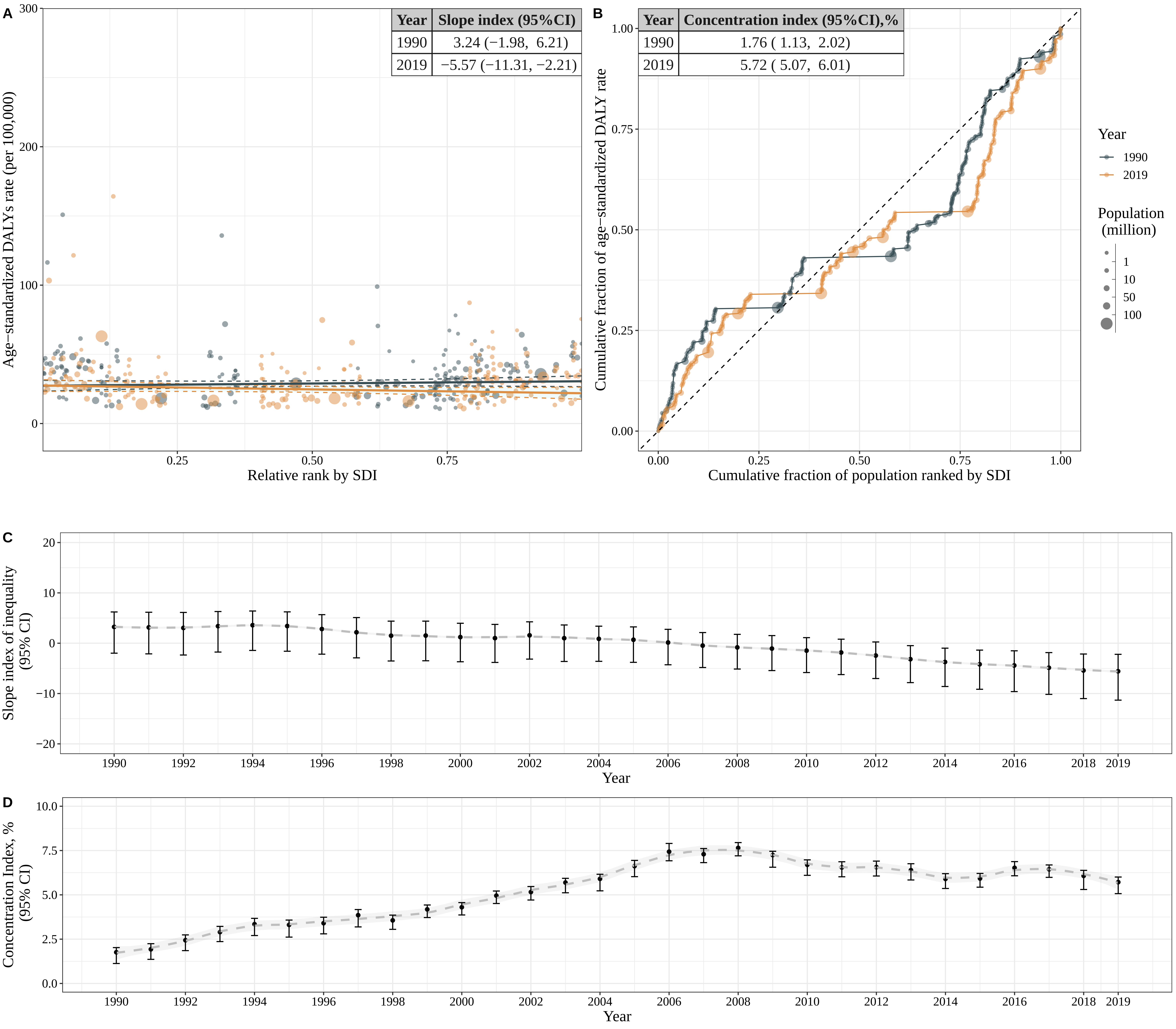
A
Year | Slope index (95%CI)
1990 | 3.24 (−1.98, 6.21)
2019 | −5.57 (−11.31, −2.21)
Age−standardized DALYs rate (per 100,000)
Relative rank by SDI
B
Year | Concentration index (95%CI),%
1990 | 1.76 (1.13, 2.02)
2019 | 5.72 (5.07, 6.01)
Cumulative fraction of age−standardized DALY rate
Cumulative fraction of population ranked by SDI
Year
1990
2019
Population (million)
1
10
50
100
C
Slope index of inequality (95% CI)
Year
D
Concentration Index, % (95% CI)
Year

Supplementary figure 4 Age standardized DALYs rates of kidney cancer in 1990, 2019 and change from 1990 to 2019. (A) Change in age standardized DALYs rates from 1990 to 2019; (B) Age standardized DALYs rates in 2019; (C) Age standardized DALYs rates in 1990;

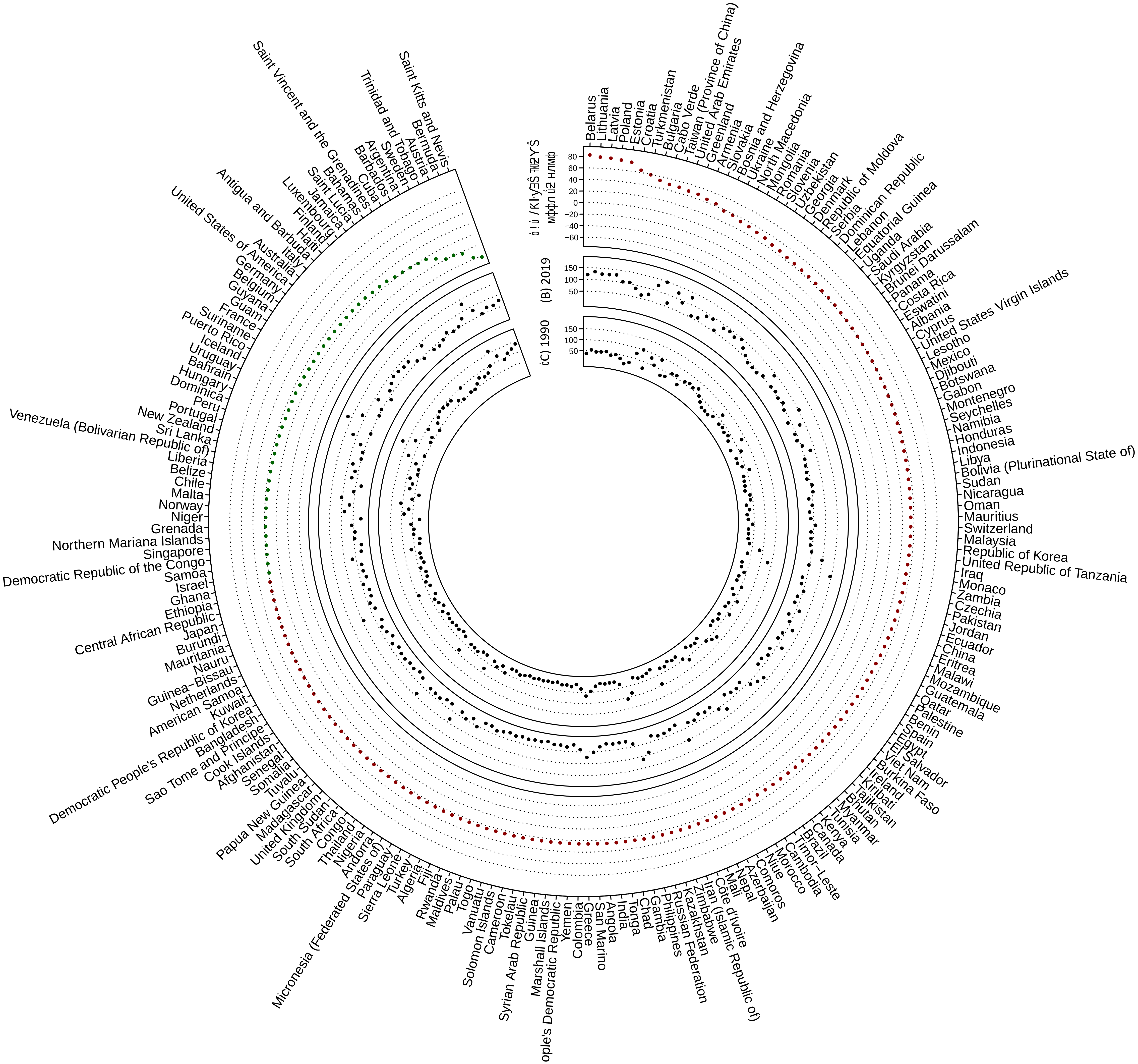

(B) 2019
(C) 1990
80
60
40
20
0
−20
−40
−60
150
100
50
150
100
50
Belarus
Lithuania
Latvia
Poland
Estonia
Croatia
Turkmenistan
Bulgaria
Cabo Verde
Taiwan (Province of China)
United Arab Emirates
Greenland
Armenia
Slovakia
Bosnia and Herzegovina
Ukraine
North Macedonia
Mongolia
Romania
Slovenia
Uzbekistan
Georgia
Denmark
Serbia
Republic of Moldova
Dominican Republic
Lebanon
Equatorial Guinea
Uganda
Saudi Arabia
Kyrgyzstan
Brunei Darussalam
Panama
Costa Rica
Eswatini
Albania
United States Virgin Islands
Cyprus
Lesotho
Mexico
Djibouti
Botswana
Gabon
Montenegro
Seychelles
Namibia
Honduras
Indonesia
Libya
Bolivia (Plurinational State of)
Sudan
Nicaragua
Oman
Mauritius
Switzerland
Malaysia
Republic of Korea
United Republic of Tanzania
Iraq
Monaco
Zambia
Czechia
Pakistan
Jordan
Ecuador
China
Eritrea
Malawi
Mozambique
Guatemala
Qatar
Palestine
Benin
Spain
Egypt
El Salvador
Viet Nam
Burkina Faso
Ireland
Kiribati
Tajikistan
Bhutan
Myanmar
Tunisia
Kenya
Canada
Brazil
Timor−Leste
Cambodia
Morocco
Niue
Comoros
Azerbaijan
Nepal
Mali
Côte d'Ivoire
Iran (Islamic Republic of)
Zimbabwe
Kazakhstan
Russian Federation
Philippines
Gambia
Chad
Tonga
India
Angola
San Marino
Greece
Colombia
Yemen
ople's Democratic Republic
Marshall Islands
Guinea
Syrian Arab Republic
Tokelau
Cameroon
Solomon Islands
Vanuatu
Togo
Palau
Maldives
Rwanda
Fiji
Algeria
Turkey
Sierra Leone
Paraguay
Micronesia (Federated States of)
Andorra
Nigeria
Thailand
Congo
South Africa
South Sudan
United Kingdom
Madagascar
Papua New Guinea
Tuvalu
Somalia
Senegal
Afghanistan
Cook Islands
Sao Tome and Principe
Bangladesh
Democratic People's Republic of Korea
Kuwait
American Samoa
Netherlands
Guinea−Bissau
Nauru
Mauritania
Burundi
Japan
Central African Republic
Ethiopia
Ghana
Israel
Samoa
Democratic Republic of the Congo
Singapore
Northern Mariana Islands
Grenada
Niger
Norway
Malta
Chile
Belize
Liberia
Venezuela (Bolivarian Republic of)
Sri Lanka
New Zealand
Portugal
Peru
Dominica
Hungary
Bahrain
Uruguay
Iceland
Puerto Rico
Suriname
France
Guam
Guyana
Belgium
Germany
United States of America
Australia
Italy
Antigua and Barbuda
Haiti
Finland
Luxembourg
Jamaica
Saint Lucia
Bahamas
Saint Vincent and the Grenadines
Cuba
Barbados
Argentina
Sweden
Trinidad and Tobago
Austria
Bermuda
Saint Kitts and Nevis

Supplementary figure 5 Inequality of kidney cancer burden in males from 1990 to 2019. (A) Scatter plot of age standardized DALYs rates and Slope index of inequality in 1990 and 2019; (B)Lorenz curve and Concentration index in 1990 and 2019; (C) Change of slope index of inequality from 1990 to 2019; (D) Change of concentration index from 1990 to 2019;

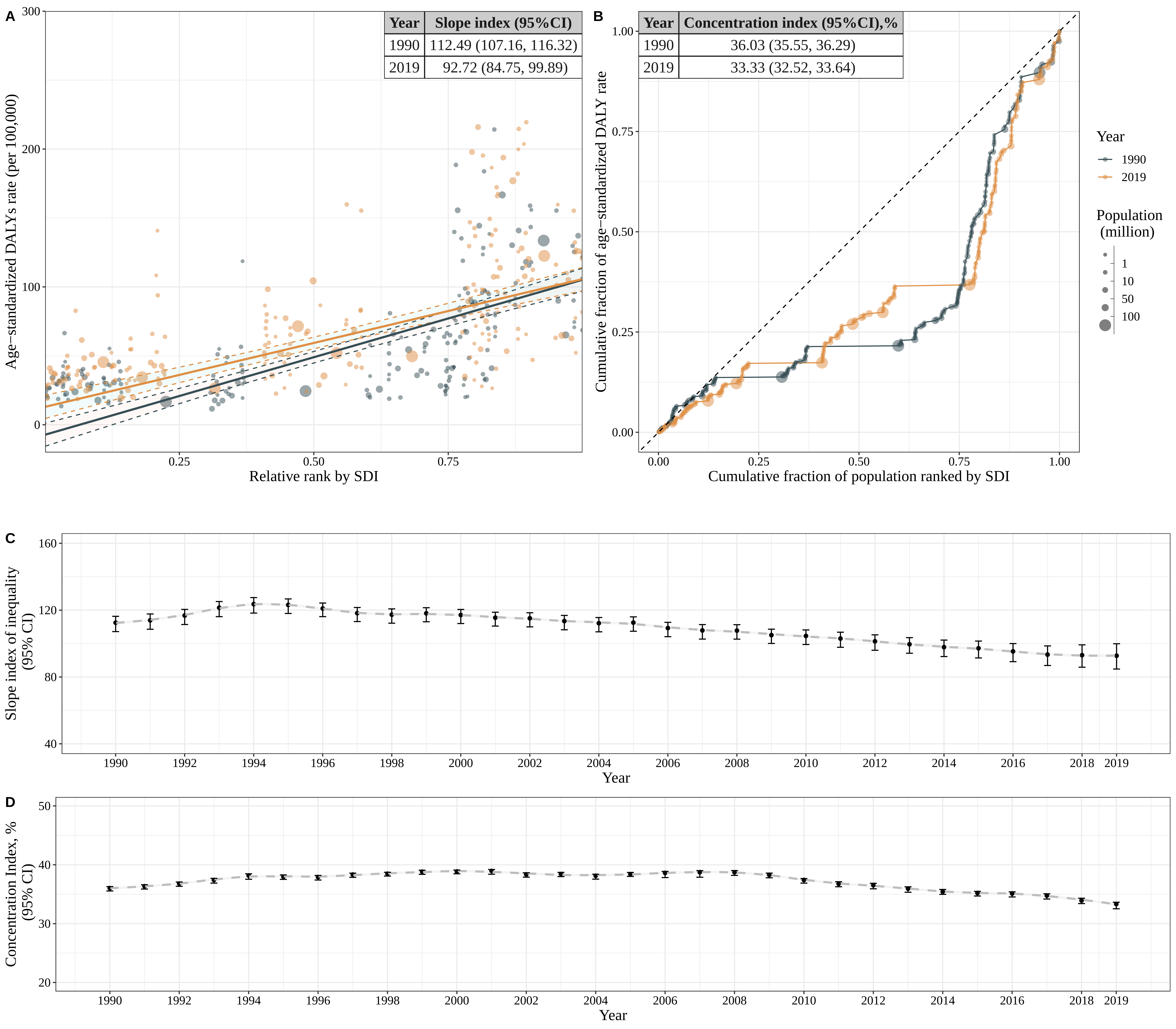
A
Year | Slope index (95%CI)
1990 | 112.49 (107.16, 116.32)
2019 | 92.72 (84.75, 99.89)
Age−standardized DALYs rate (per 100,000)
Relative rank by SDI
B
Year | Concentration index (95%CI),%
1990 | 36.03 (35.55, 36.29)
2019 | 33.33 (32.52, 33.64)
Cumulative fraction of age−standardized DALY rate
Cumulative fraction of population ranked by SDI
Year
1990
2019
Population (million)
1
10
50
100
C
Slope index of inequality (95% CI)
Year
D
Concentration Index, % (95% CI)
Year

Supplementary figure 6 Inequality of kidney cancer burden in females from 1990 to 2019. (A) Scatter plot of age standardized DALYs rates and Slope index of inequality in 1990 and 2019; (B)Lorenz curve and Concentration index in 1990 and 2019; (C) Change of slope index of inequality from 1990 to 2019; (D) Change of concentration index from 1990 to 2019;

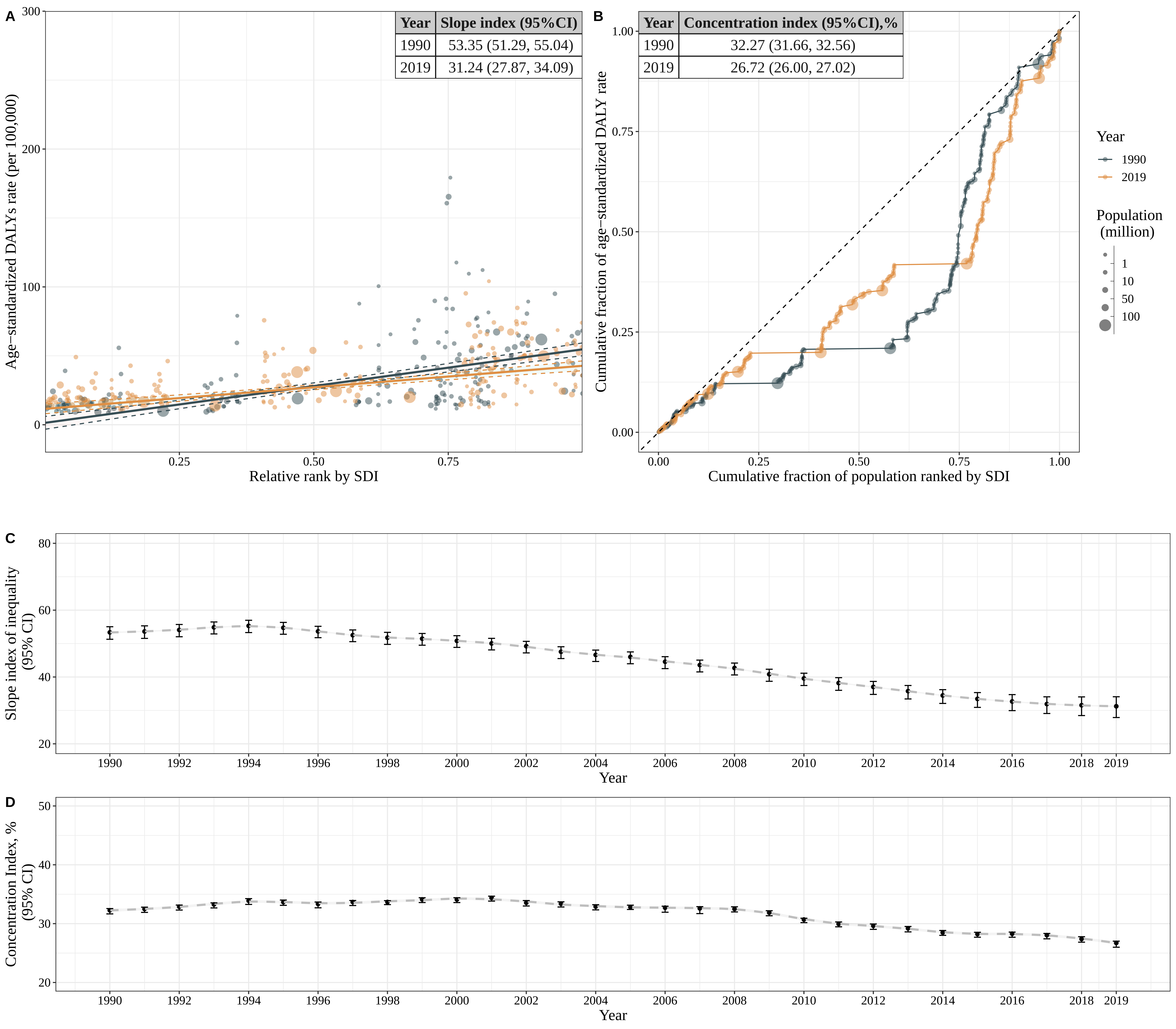
A
Year | Slope index (95%CI)
1990 | 53.35 (51.29, 55.04)
2019 | 31.24 (27.87, 34.09)
Age−standardized DALYs rate (per 100,000)
Relative rank by SDI
B
Year | Concentration index (95%CI),%
1990 | 32.27 (31.66, 32.56)
2019 | 26.72 (26.00, 27.02)
Cumulative fraction of age−standardized DALY rate
Cumulative fraction of population ranked by SDI
Year
1990
2019
Population (million)
1
10
50
100
C
Slope index of inequality (95% CI)
Year
D
Concentration Index, % (95% CI)
Year

Supplementary figure 7 Age standardized DALYs rates of prostate cancer in 1990, 2019 and change from 1990 to 2019. (A) Change in age standardized DALYs rates from 1990 to 2019; (B) Age standardized DALYs rates in 2019; (C) Age standardized DALYs rates in 1990;

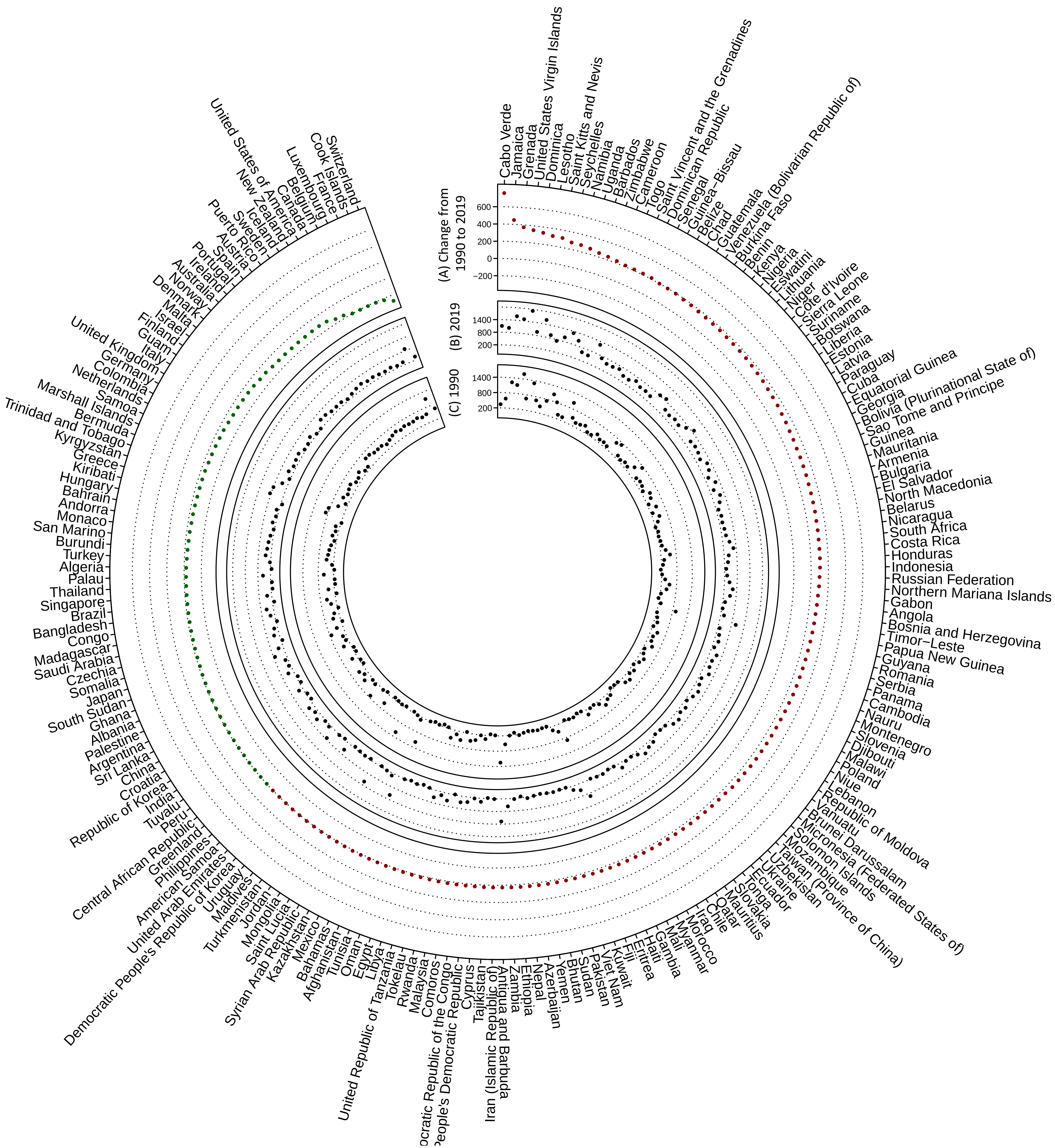
(A) Change from 1990 to 2019
600
400
200
0
−200
(B) 2019
1400
800
200
(C) 1990
1400
800
200
Cabo Verde
Jamaica
Grenada
United States Virgin Islands
Dominica
Lesotho
Saint Kitts and Nevis
Seychelles
Namibia
Uganda
Barbados
Zimbabwe
Cameroon
Togo
Saint Vincent and the Grenadines
Dominican Republic
Senegal
Guinea−Bissau
Belize
Chad
Guatemala
Venezuela (Bolivarian Republic of)
Burkina Faso
Benin
Kenya
Nigeria
Eswatini
Lithuania
Niger
Cote d'Ivoire
Sierra Leone
Suriname
Botswana
Liberia
Estonia
Latvia
Paraguay
Cuba
Equatorial Guinea
Georgia
Bolivia (Plurinational State of)
Sao Tome and Principe
Guinea
Mauritania
Armenia
Bulgaria
El Salvador
North Macedonia
Belarus
Nicaragua
South Africa
Costa Rica
Honduras
Indonesia
Russian Federation
Northern Mariana Islands
Gabon
Angola
Bosnia and Herzegovina
Timor−Leste
Papua New Guinea
Guyana
Romania
Serbia
Panama
Cambodia
Nauru
Montenegro
Slovenia
Djibouti
Malawi
Poland
Niue
Lebanon
Republic of Moldova
Vanuatu
Brunei Darussalam
Micronesia (Federated States of)
Solomon Islands
Mozambique
Taiwan (Province of China)
Uzbekistan
Ukraine
Ecuador
Tonga
Slovakia
Mauritius
Qatar
Chile
Iraq
Morocco
Myanmar
Mali
Gambia
Haiti
Eritrea
Fiji
Kuwait
Viet Nam
Pakistan
Sudan
Bhutan
Yemen
Azerbaijan
Nepal
Ethiopia
Zambia
Antigua and Barbuda
Iran (Islamic Republic of)
Tajikistan
Cyprus
eople's Democratic Republic
ocratic Republic of the Congo
Comoros
Malaysia
Rwanda
Tokelau
United Republic of Tanzania
Libya
Egypt
Oman
Tunisia
Afghanistan
Bahamas
Mexico
Kazakhstan
Syrian Arab Republic
Saint Lucia
Mongolia
Jordan
Turkmenistan
Maldives
Uruguay
Democratic People's Republic of Korea
United Arab Emirates
American Samoa
Philippines
Greenland
Central African Republic
Peru
Tuvalu
Republic of Korea
India
Croatia
China
Sri Lanka
Argentina
Palestine
Albania
Ghana
South Sudan
Japan
Somalia
Czechia
Saudi Arabia
Madagascar
Congo
Bangladesh
Brazil
Singapore
Thailand
Palau
Algeria
Turkey
Burundi
San Marino
Monaco
Andorra
Bahrain
Hungary
Kiribati
Greece
Kyrgyzstan
Trinidad and Tobago
Bermuda
Marshall Islands
Samoa
Netherlands
Colombia
Germany
United Kingdom
Italy
Guam
Finland
Israel
Malta
Denmark
Norway
Australia
Ireland
Portugal
Spain
Austria
Puerto Rico
Sweden
Iceland
New Zealand
United States of America
Canada
Belgium
Luxembourg
France
Cook Islands
Switzerland

Supplementary figure 8 Age standardized DALYs rates of bladder, kidney and prostate cancer in 2019 along with sociodemographic index. (A) Sociodemographic index; (B) Age-standardized DALY rate of bladder cancer; (C) Age-standardized DALY rate of kidney cancer; (D)Age-standardized DALY rate of prostate cancer;

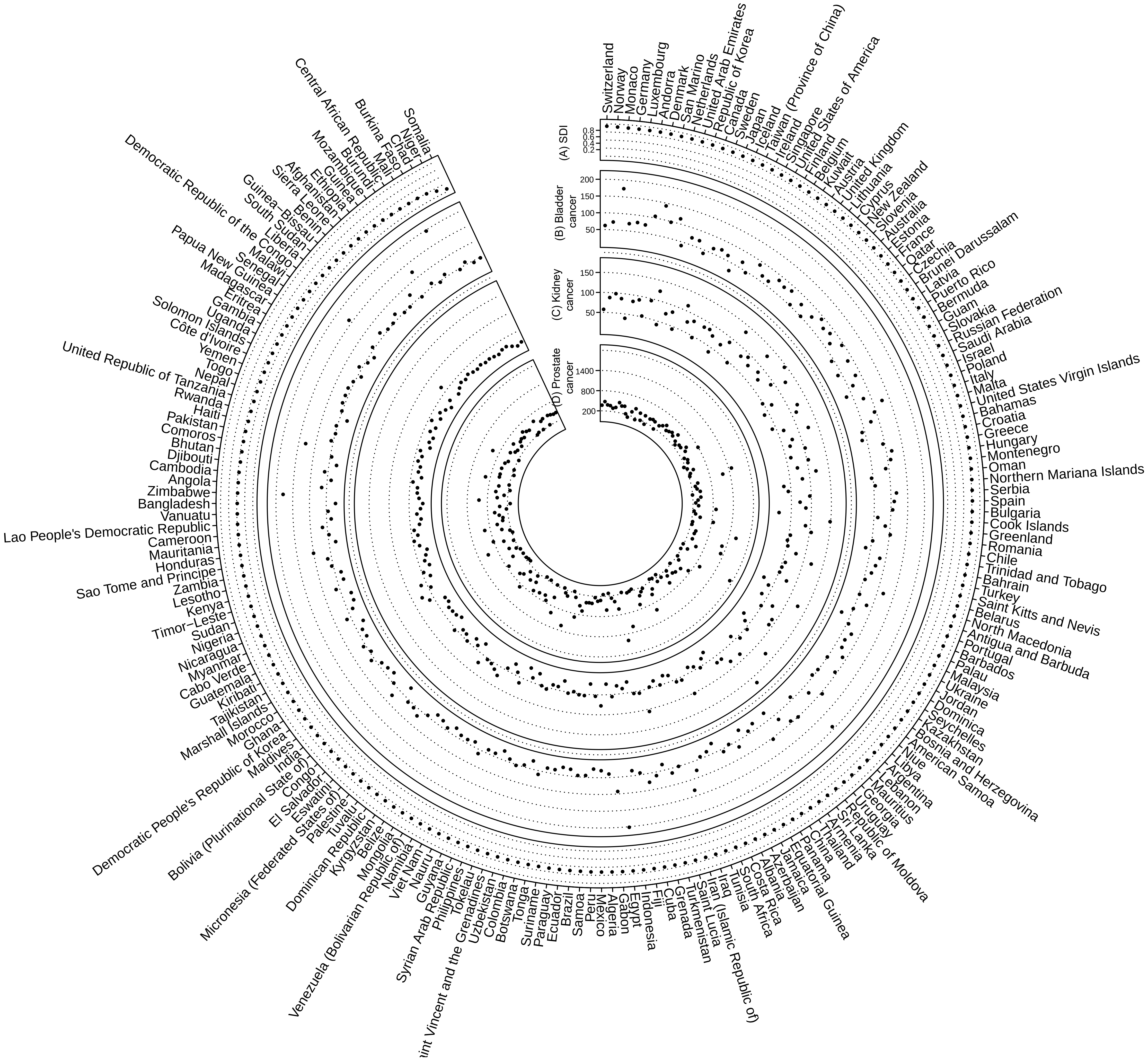
(A) SDI
0.8
0.6
0.4
0.2
(B) Bladder cancer
200
150
100
50
(C) Kidney cancer
150
100
50
(D) Prostate cancer
1400
800
200
Switzerland
Norway
Monaco
Germany
Luxembourg
Andorra
Denmark
San Marino
Netherlands
United Arab Emirates
Republic of Korea
Canada
Sweden
Japan
Iceland
Taiwan (Province of China)
Ireland
Singapore
United States of America
Finland
Belgium
Kuwait
Austria
United Kingdom
Lithuania
Cyprus
New Zealand
Slovenia
Australia
Estonia
France
Qatar
Czechia
Brunei Darussalam
Latvia
Puerto Rico
Bermuda
Guam
Slovakia
Russian Federation
Saudi Arabia
Israel
Poland
Italy
Malta
United States Virgin Islands
Bahamas
Croatia
Greece
Hungary
Montenegro
Oman
Northern Mariana Islands
Serbia
Spain
Bulgaria
Cook Islands
Greenland
Romania
Chile
Trinidad and Tobago
Bahrain
Turkey
Saint Kitts and Nevis
Belarus
North Macedonia
Antigua and Barbuda
Portugal
Barbados
Palau
Malaysia
Ukraine
Jordan
Dominica
Seychelles
Kazakhstan
Bosnia and Herzegovina
American Samoa
Niue
Libya
Argentina
Lebanon
Mauritius
Georgia
Uruguay
Republic of Moldova
Sri Lanka
Armenia
Thailand
China
Panama
Equatorial Guinea
Jamaica
Azerbaijan
Albania
Costa Rica
South Africa
Tunisia
Iraq
Iran (Islamic Republic of)
Saint Lucia
Turkmenistan
Grenada
Cuba
Fiji
Indonesia
Egypt
Gabon
Algeria
Mexico
Peru
Samoa
Brazil
Ecuador
Paraguay
Suriname
Tonga
Botswana
Colombia
Uzbekistan
aint Vincent and the Grenadines
Tokelau
Philippines
Syrian Arab Republic
Guyana
Nauru
Viet Nam
Namibia
Venezuela (Bolivarian Republic of)
Mongolia
Belize
Kyrgyzstan
Dominican Republic
Tuvalu
Micronesia (Federated States of)
Palestine
Eswatini
El Salvador
Bolivia (Plurinational State of)
Congo
India
Democratic People's Republic of Korea
Maldives
Ghana
Morocco
Marshall Islands
Tajikistan
Kiribati
Guatemala
Cabo Verde
Myanmar
Nicaragua
Nigeria
Sudan
Timor−Leste
Kenya
Lesotho
Zambia
Sao Tome and Principe
Honduras
Mauritania
Cameroon
Lao People's Democratic Republic
Vanuatu
Bangladesh
Zimbabwe
Angola
Cambodia
Djibouti
Bhutan
Comoros
Pakistan
Haiti
Rwanda
United Republic of Tanzania
Nepal
Togo
Yemen
Côte d'Ivoire
Solomon Islands
Uganda
Gambia
Eritrea
Madagascar
Papua New Guinea
Senegal
Malawi
Democratic Republic of the Congo
Liberia
South Sudan
Guinea−Bissau
Benin
Sierra Leone
Afghanistan
Ethiopia
Guinea
Mozambique
Burundi
Central African Republic
Mali
Burkina Faso
Chad
Niger
Somalia
